# Optimal satellite selection using quantum convolutional autoencoder for low-cost GNSS receiver applications

**DOI:** 10.1038/s41598-025-91959-0

**Published:** 2025-03-13

**Authors:** Nalineekumari Arasavali, Mogadala Vinod Kumar, Sasibhushana Rao Gottapu, Om Prakash Kumar, Priyanka Desai Kakade, Shweta Vincent, Pallavi R. Mane, Sachin Kumar

**Affiliations:** 1 Department of Electronics & Communication Engineering, Dadi Institute of Engineering & Technology(A), Visakhapatnam, Andhra Pradesh India; 2 Department of Electronics & Communication Engineering, Dhanekula Institute of Engineering & Technology(A), Vijayawada, Andhra Pradesh India; 3 Department of Electronics & Communication Engineering, Andhra university College of Engineering(A), Visakhapatnam, Andhra Pradesh India; 4 Department of Electronics and Communication Engineering, Manipal Institute of Technology, Manipal Academy of Higher Education, Manipal, 576104 India; 5 School of Engineering and Built Environment, Sheffield Hallam University, Sheaf Building, City Campus, Sheffield, S1 1WB UK; 6 Department of Mechatronics, Manipal Institute of Technology, Manipal Academy of Higher Education, Manipal, 576104 India; 7 Department of Electronics and Communication Engineering, Galgotias College of Engineering and Technology, Greater Noida, 201310 India

**Keywords:** Satellite selection, Quantum convolutional autoencoder, GNSS, Combined constellation, Sustainable Development Goal (SDG) 9:Industry, Innovation, and Infrastructure, SDG 11: Sustainable Cities and Communities, Engineering, Electrical and electronic engineering

## Abstract

The increasing reliance on global navigation satellite systems for diverse applications necessitates the development of efficient satellite selection methods to optimize positioning accuracy and system performance. In particular, low-cost global navigation satellite systems receivers face challenges in managing data from multiple visible satellites, often resulting in suboptimal performance due to high geometric dilution of precision values. Effective satellite selection is crucial for improving the accuracy and reliability of positioning solutions in these systems. Quantum computing and machine learning provide promising solutions by using data patterns for complex optimization problems. This work proposes the quantum convolutional autoencoder-based optimal satellite selection method. This new satellite selection method examined the data collected from the receiver located at latitude 16.33° N and longitude 80.62° E, collected on March 10, 2022. The main aim is to enhance the performance of low-cost receivers by minimizing the geometric dilution of precision values and optimizing the tetrahedron volume function. Quantum convolutional autoencoders process the satellite data to balance the navigational solution’s computational burden and the navigational algorithm’s accuracy. The model aims to identify the most optimal satellites for positioning by setting geometric dilution of precision as the cost function. The QCAE-based method achieves a CEP of 1.384 m and SEP of 1.759 m for four selected satellites, compared to 5.937 m and 6.691 m for PSOSSM. For nine satellites, QCAE achieves a CEP of 1.287 m and SEP of 1.713 m, while PSOSSM results in 5.725 m and 6.385 m, respectively. Additionally, QCAE reduces computations by over 64%, requiring 730 multiplications and 713 additions, compared to 2034 multiplications and 2017 additions for all visible satellites. This proposed approach provides the optimal navigation solution for cost-effective implementations in a real-time environment. This research provides new insights into satellite selection strategies using machine learning approaches.

## Introduction

Global Navigation Satellite Systems (GNSS) are useful for navigation, mapping, and surveying. The effectiveness in terms of accuracy of GNSS navigation depends on the position of satellites at the same time it is very important to select optimal subset of satellites to ensure high positioning accuracy while managing computational efficiency for low cost GNSS receiver applications. As GNSS technology advances and the number of satellites increases, traditional methods of satellite selection face significant challenges related to computational complexity and real-time performance. Traditional methods for satellite selection predominantly involve mathematical and geometric criteria. The basic navigation solution, least squares approach consists solving a system of linear equations to estimate the receiver’s position based on satellite observations.

Though it is effective, the least squares method becomes computationally intensive with an increasing number of satellites. As the volume of geometry grows, the computational burden also increases and that affects the system’s ability to deliver real-time performance^1–3^. The least squares method is proved as a best method to minimize the sum of squared residuals between observed and predicted measurements, but the complexity of mathematical calculations can impede its practical application in high-density satellite environments^4,5^. GDOP decides the impact of satellite geometry on positioning accuracy, with lower GDOP values indicating better satellite configurations. Similarly, maximizing tetrahedron volume aims to select satellites that provide the most favourable geometric arrangement for accurate positioning^6,7^. However, these methods are not suitable when dealing with dynamic satellite configurations as the computational efficiency will increase the burden on navigational solution as well as on the GNSS receiver. They focus primarily on accuracy rather than the computational burden associated with processing of information associated with the satellites^8,9^. Various optimization algorithms like Genetic Algorithms (GAs) and Particle Swarm Optimization (PSO) have been explored. Genetic Algorithm utilises evolutionary techniques to iteratively select the best satellite configurations based on fitness functions, while PSO, inspired by swarm intelligence, optimizes configurations by evaluating potential solutions and adjusting based on collective swarm behaviour^10–13^. Although these algorithms provided the improved performance, but their effectiveness is limited by their inability to fully adapt to real-time changes in satellite visibility and the complexity of the optimization process^14,15^. The integration of machine learning, particularly quantum machine learning methods, introduces a new approach in satellite selection. Convolutional Autoencoders (CAEs) and Quantum Convolutional Autoencoders (QCAEs) proved their potential in modelling complex relationships and making predictions based on large datasets. Generally, QCAEs use the quantum computing features to handle high-dimensional data efficiently. By processing data through quantum layers, QCAEs can optimize satellite selection while maintaining high positioning accuracy^16,17^. In general, QCAEs used to learn from historical data to understand the dynamic conditions and to provide a flexible and efficient approach to satellite selection. Recent research studies are explaining the performance of quantum machine learning techniques in classification tasks and these are outer performing the traditional methods and optimization techniques in both accuracy and computational efficiency^18^. Neish et al.^19^ explored quantum-resistant authentication algorithms for satellite‐based augmentation systems for predicting satellite visibility, achieving notable performance improvements. Duan et al.^20^ explained the quantum positioning, showing that quantum machine learning methods could provide superior results compared to conventional approaches in various scenarios. These studies underscore the potential of quantum machine learning in enhancing GNSS performance, addressing both accuracy and computational challenges. Further advancements have been made in this field. Calderaro et al.^21^ explored the quantum communication from global navigation satellite systems, focusing on reducing computational complexity while maintaining high accuracy. Feng, explained the quantumm navigation procedures and the application of quantum techniques to GNSS navigation, highlighting the advantages of these methods in addressing computational challenges^22^. Lesouple et al.^23^ investigated the use of quantum reinforcement learning for positioning and navigation applications, demonstrating improved performance over traditional optimization methods. These contributions show the effectiveness of quantum machine learning in GNSS navigation. The use of QCAEs may give the feasible solution for multi-constellation satellite navigation systems. QCAEs can learn from large datasets and adapt to dynamic satellite visibility changes, making satellite selection flexible and efficient. Unlike older approaches, QCAEs can reduce satellites needed for precise positioning without compromising performance. QCAEs can choose appropriate satellite subsets to maintain positioning accuracy by training on past data. This method decreases computational load and maintains positioning performance with all visible satellites. Based on this foundation, the proposed research implements QCAEs for satellite selection in GNSS systems to minimize computational burden and maintain performance. The study will use data from a GNSS receiver tracking GLONASS and GPS satellites to show that optimal satellite selection using QCAEs can achieve the same positioning performance as all visible satellites. Finally, adding QCAEs to satellite selection processes advances GNSS technology. QCAEs improve satellite selection efficiency and flexibility by addressing the constraints of previous approaches and optimization algorithms.

This work introduces a Quantum Convolutional Autoencoder (QCAE)-based satellite selection method that optimizes geometric dilution of precision (GDOP). The primary distinguishing factor of the Quantum Convolutional Autoencoder (QCAE)-based satellite selection method is its superior capability to maximize both the geometric dilution of precision (GDOP) and the Tetrahedron Volume function compared to current methodologies. Conventional approaches typically concentrate on a certain optimization metric or entail increased processing complexity. Conversely, the QCAE technique utilizes the intrinsic parallelism of quantum computing to alleviate the computational load while preserving or enhancing positioning precision. This method not only reduces GDOP but also facilitates effective satellite selection, rendering it very beneficial for economical GNSS receivers. This work also pioneers the use of quantum computing techniques like QCAE to pick satellites, presenting a possible solution for complicated GNSS optimization challenges. The suggested technique is validated with real-world GNSS data from a receiver at 16.33° N and 80.62° E on March 10, 2022, proving its practicality and performance enhancement. The QCAE-based technique balances computational burden and performance, making real-time satellite selection cost-effective.

Further, section “Methodology” describes the methodology, including how the QCAE-based system processes GNSS data to reduce GDOP and optimize satellite selection. Section “Results” analyses the experimental results, which compare the suggested method’s accuracy, specificity, and computational efficiency to that of current procedures. Finally, section “Conclusion” concludes by reviewing the advantages of the proposed method and noting potential future applications with respect to multi-constellation GNSS systems.

## Methodology

In general, to compute position, a GNSS receiver calculates its distance to visible satellites^24^. The receiver calculates the distance by measuring the time δt, it takes to travel from satellite to receiver and this distance is called as pseudo-range (P) in Eq. (1).

1 $$p=\delta t.c$$

Where, ‘c’ is the speed of light in free space. The range between user and ith satellite is calculated as given in Eq. (2).

2 $$p=\sqrt {{{\left( {{X_{sati}} - X} \right)}^2}+{{\left( {{Y_{sati}} - Y} \right)}^2}+{{\left( {{Z_{sati}} - Z} \right)}^2}}$$

Where, *(X*,* Y*,* Z)* indicates the user position and $\left( {{{\text{X}}_{{\text{sati}}}},{{\text{Y}}_{{\text{sati}}}},{{\text{Z}}_{{\text{sati}}}}} \right)$ gives the ith satellite position. The observed pseudo-range is the combination of modelled pseudo-range, process noise and system errors are expressed in Eq. (3).

3 $${p_{observed}}={p_{Computed}}+Noise$$

$$=p\left( {x,y,z,t} \right)+v$$

The residual observation is given in Eq. (3), difference between the p_observed_ and p_Computed_ can be represented in matrix form for ‘m’ number of satellites as shown in Eq. (4)

$$\Delta P={P_{observed}} - {P_{Computed}}$$

4 $$\left[ {\begin{array}{*{20}c} {\begin{array}{*{20}c} {\Delta P^{1} } \\ {\Delta P^{2} } \\ {\Delta P^{3} } \\ \end{array} } \\ \vdots \\ {\Delta P^{m} } \\ \end{array} } \right] = \left[ {\begin{array}{*{20}c} {\frac{{\partial P^{1} }}{{\partial x}}} & {\frac{{\partial P^{1} }}{{\partial y}}} & {\frac{{\partial P^{1} }}{{\partial z}}} & {\frac{{\partial P^{1} }}{{\partial \tau }}} \\ {\frac{{\partial P^{2} }}{{\partial x}}} & {\frac{{\partial P^{2} }}{{\partial y}}} & {\frac{{\partial P^{2} }}{{\partial z}}} & {\frac{{\partial P^{2} }}{{\partial \tau }}} \\ {\frac{{\partial P^{3} }}{{\partial x}}} & {\frac{{\partial P^{3} }}{{\partial y}}} & {\frac{{\partial P^{3} }}{{\partial z}}} & {\frac{{\partial P^{3} }}{{\partial \tau }}} \\ \vdots & \vdots & \vdots & \vdots \\ {\frac{{\partial P^{m} }}{{\partial x}}} & {\frac{{\partial P^{m} }}{{\partial y}}} & {\frac{{\partial P^{m} }}{{\partial z}}} & {\frac{{\partial P^{m} }}{{\partial \tau }}} \\ \end{array} } \right]\left[ {\begin{array}{*{20}c} {\Delta x} \\ {\Delta y} \\ {\Delta z} \\ {\Delta \tau } \\ \end{array} } \right] + \left[ {\begin{array}{*{20}c} {v^{1} } \\ {v^{2} } \\ {v^{3} } \\ \vdots \\ {v^{m} } \\ \end{array} } \right]$$

The Eq. (4) is often written in terms of matrix symbols (b- Residuals, Gd-Design matrix, v-Noise terms) as

5 $${\text{b}}\,=\,{\text{G}}\_{\text{d X}}\,+\,{\text{v}}$$

Observation matrix G_d is purely a function of the direction to each of the satellites as observed from the receiver.

6 $${G_d}=\left[ {\begin{array}{*{20}{c}} {{j_1}}&{{k_1}}&{{l_1}}&{ - 1} \\ {{j_2}}&{{k_2}}&{{l_2}}&{ - 1} \\ {{j_3}}&{{k_3}}&{{l_3}}&{ - 1} \\ \vdots & \vdots & \vdots & \vdots \\ {{j_n}}&{{k_n}}&{{l_n}}&{ - 1} \end{array}} \right]$$

J_i_, k_i_ and l_i_ are three components of satellite Si. A least squares solution is as follows

7 $$X={\left( {{G_d}^{T}{G_d}} \right)^{ - 1}}{G_d}^{T}b$$

The error in estimation depends on residual measurement and clock bias errors and is considered as

8 $$\Delta X=~{\left[ {\Delta {r^T}\Delta b} \right]^T}$$

The covariance of position is given by

9 $$E=~\widehat {{\Delta X}}\widehat {{\Delta {X^T}}}=~{\sigma ^2}{\left( {{G_d}^{T}{G_d}} \right)^{ - 1}}$$

The matrix ${\left( {{G_d}^{T}{G_d}} \right)^{ - 1}}~$represents GDOP matrix and scalar value of GDOP is obtained by taking square root of the trace of the GDOP matrix^25,26^.

10 $$GDOP=~\sqrt {trace{{\left( {{G_d}^{T}{G_d}} \right)}^{ - 1}}}$$

The accuracy of least square solution is decided by measurement quality and satellite receiver geometry. Measurement quality is investigated by the factor σ2 and geometry is given by values present in design matrix. The change of GDOP is always depends on number of satellites. The GDOP is always decreases in a nonlinear way when number satellites increase^25–33^.

And how the number of satellites is affecting the computations involved in a basic positioning navigation solution are discussed below. The relationship between the number of multiplications and additions and the number of satellites is given below (Eqs. 11 and 12).

Number of multiplications:

11 $$\left( {1/3} \right)I_{s}^{2} + \left( {N + 1} \right)I_{s}^{2} + \left( {N^{2} + N - \left( {1/3} \right)} \right)I_{s}^{2} + 3N$$

Number of Additions:

12 $$\left( {1/3} \right)I_{s}^{2} + \left( {N + \left( {1/2} \right)} \right)I_{s}^{2} + \left( {N^{2} + N - \left( {11/6} \right)I_{s} } \right) + 3N$$

**Fig. 1:**
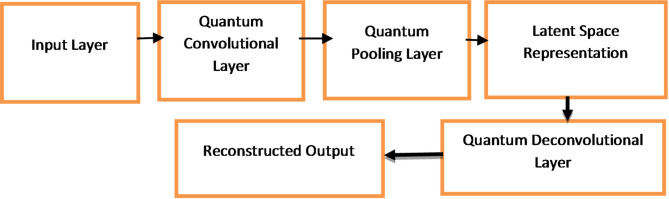
Block diagram of QCAE.

Where, N represents the number of selected satellites and Is = 3 + S where S is number of navigation systems. If the number of satellites used in position calculations increases, the computational burden also increases. Thus, navigation computation is related to number of selected satellites. Hence optimal satellite selection is essential for low cost GNSS receivers. The proposed technique, QACE, is analysed using data from an epoch on March 10, 2022, at a location with a latitude of 16.33° N and longitude of 80.62° E. At that epoch the visible satellites are Satellite Numbers: 24, 14, 29, 20, 21, R20, R21, R05, R18, R19, R15, 15, 32, R06, 10, R04, 27, the number preceding with R is representing the GLONASS satellites. The GNSS receiver at these coordinates can receive data from both GPS and GLONASS satellites, as combined constellations enhance positioning accuracy. Each satellite provides information such as Satellite ID, Position (latitude, longitude, altitude), Signal strength (SNR), and Time of observation. In the data preprocessing stage, the satellite position data needs to be normalized to extract features such as Satellite position, SNR, and other relevant parameters. The QCAE model consists of quantum convolutional layers that extract spatial features from satellite data and quantum pooling layers that reduce dimensionality while preserving essential information. The training process employs the Adam optimizer with a learning rate of 0.001, batch size of 32, and 50 epochs. The detailed architecture is described as follows:

The Quantum Convolutional Autoencoder (QCAE) Architecture, as shown in Fig. 1, consists of Encoder and Decoder^34^. The encoder consists of quantum convolutional layer and quantum pooling layer. The quantum convolutional layer applies a set of quantum filters to the input data. The output of quantum convolutional layer can be represented as

13 $$Convolution~Output=f\left. {\left( {W.X+b} \right)} \right)$$

Where, W represents the quantum convolutional weights, X is the input data and b is the bias term.

The input data matrix for the convolutional layer at the given epoch includes the visible satellites with the following Satellite Numbers: 24, 14, 29, 20, 21, R20, R21, R05, R18, R19, R15, 15, 32, R06, 10, R04, 27.

14 $$X=\left[ {\begin{array}{*{20}{c}} {{x_{24}}}&{{y_{24}}}&{\begin{array}{*{20}{c}} {{z_{24}}}&{SN{R_{24}}} \end{array}} \\ {{x_{14}}}&{{y_{14}}}&{\begin{array}{*{20}{c}} {{z_{14}}}&{SN{R_{14}}} \end{array}} \\ {\begin{array}{*{20}{c}} \vdots \\ {{x_{27}}} \end{array}}&{\begin{array}{*{20}{c}} \vdots \\ {{y_{27}}} \end{array}}&{\begin{array}{*{20}{c}} {\begin{array}{*{20}{c}} \vdots \\ {{z_{27}}} \end{array}}&{\begin{array}{*{20}{c}} \vdots \\ {SN{R_{27}}} \end{array}} \end{array}} \end{array}} \right]$$

Where x, y & z are the position coordinates and SNR represents the Signal to Noise Ratio.

By combining the most important features identified by the convolutional layer, the Quantum Pooling layer lowers the number of dimensions in the data. The output of this layer can be represented as

15 $$Pooled~Output=\hbox{max} \left( {~Convolutional~Output} \right)$$

The output of the quantum convolutional and pooling layers creates the latent space representation, which is a compressed version of the original input that maintains important information. This representation is a lower-dimensional vector including the important properties required for optimal satellite selection. The latent space simplifies the incoming data, making it easier to handle and evaluate. By focusing on important parameters include the geometric configuration of satellites, signal strength, and visibility factors like elevation angles, all of which are essential for optimizing positioning accuracy, it eliminates duplicated or irrelevant data, increasing computing efficiency. The latent space captures the underlying patterns in satellite data, such as geometric configurations and signal levels, which are critical to location accuracy. It enables the QCAE to learn the ideal satellite configurations for various epochs. Using the latent space representation, the QCAE can determine the optimum subsets of satellites (e.g., sets of four or nine satellites) that reduce GDOP while increasing tetrahedron volume. This decision is based on compressed features, guaranteeing that the picked satellites deliver the highest positioning accuracy with the least computing burden.

The next critical component of the QCAE design is the decoder, which is responsible for reconstructing the original input data from the compressed latent space representation. The decoder consists of quantum deconvolutional layers and maybe extra layers that expand the latent space representation back to the original data dimensions. The decoder’s output is a reconstructed version of the original input data, with the goal of being as near to the original as possible. The deconvolutional layer reverses the action of the quantum convolutional layer, enlarging the latent space representation back to a higher-dimensional space. It then utilizes a set of learnt weights to project the latent features into the original data dimensions.

16 $$Deconvolution~Output=f\left( {W^{\prime}.Z+b^{\prime}} \right)$$

Where, Z is latent space representation, W’ is deconvolutional weights and b’ is the bias term. The reconstruction loss can be calculated as the difference between the original input data and the reconstructed output. Common loss functions include Mean Squared Error (MSE) or Mean Absolute Error (MAE)^35^.

17 $$Reconstruction~Loss=~\frac{1}{N}\mathop \sum \limits_{N}^{{i=1}} ({X_i} - {\widehat {{{X_i})}}^2}$$

Where, X_i_ is the original input data and $\widehat {{{X_i}}}$ is the reconstructed data.

The training of a Quantum Convolutional Autoencoder (QCAE) starts with initializing the weights and biases^34^. In the forward pass, the input data is encoded to produce a latent space representation, which is subsequently decoded to reconstruct the original data. The reconstruction loss, which is the difference between the original input and the rebuilt output, is calculated next. During the backward pass, backpropagation is used to determine the loss gradients in relation to the model parameters.

These gradients are then used to update the model parameters via an optimization algorithm such as Adam or SGD, with the learning rate determining the step size for each update. This sequence of forward pass, loss calculation, backward pass, and parameter update is repeated iteratively for a set number of epochs or until the model converges. The proposed technique employs selection criteria that minimize GDOP and maximize tetrahedron volume to choose a subset of satellites. The comparison analysis requires GNSS accuracy measures, which are tabulated in Table 1 below.

**Table 1:** GNSS accuracy measures.

CEP (Circular Error Probable) ≈ 0.589 ⋅ σ_*r*_ Where, σ_*r*_ is the standard deviation of the radial error
SEP (Spherical Error Probable) ≈ 0.526 ⋅ σ Where, σ is the standard deviation of the 3D position error
2DRMS (2-Dimensional Root Mean Square) = 2 $\sqrt {\left( {\sigma _{x}^{2} ~ + ~\sigma _{y}^{2} } \right)}$ Where, σ_x_ and σ_y_ are the standard deviations of the position errors in the x and y coordinates, respectively
MRSE (Mean Radial Spherical Error) =$\sqrt {\left( {{\text{\varvec{\upsigma}}}_{x}^{2}{\text{~}}+{\text{~\varvec{\upsigma}}}_{y}^{2}{\text{~}}+{\text{~\varvec{\upsigma}}}_{z}^{2}} \right)}$ Where, σ_x_, σ_y_, and σ_z_ are the standard deviations of the position errors in the x, y, and z coordinates, respectively

To evaluate the performance of the proposed method, a novel satellite selection approach based on swarm intelligence, namely PSO-based Satellite Selection Method (PSOSSM), was implemented using the Particle Swarm Optimization (PSO) principle^36–43^. The PSOSSM involves six processing phases, which are as follows:

1. Extraction of observed satellites from collected navigation data.
2. Encoding: Binary encoding is preferred in PSOSSM for satellite selection.
3. Initialization of particle population.

In order to select satellites from dimensional solution space which consists of all visible satellites, search schemes need to be selected to generate initial population.

In order to select ${{\prime }}r{{\prime }}$ satellites from ${{\prime }}N{{\prime }}$ dimensional solution space which consists of all visible satellites, ${{\prime }}{S_s}{{\prime }}$ search schemes need to be selected to generate initial population.

18 $${Y^d}=~Y_{i}^{d}~,~i=1,2, \ldots ,{S_s}.$$

And the size of population is ${S_s}.Y_{i}^{d}=\left[ {S_{{i,1}}^{d}~S_{{i,2}}^{d} \ldots .S_{{i,r}}^{d}} \right]$, where $S_{{i,k}}^{d}$ is the kth satellite of particle ‘i’ in the dth search.

4. Selection of suitable fitness function

In order to get more precise optimum combinations of satellites, the fitness function used is GDOP, which is also utilised for QACE.

$$Fit=\sqrt {Tr{{\left( {\left( {{G_d} \times G_{d}^{T}} \right)} \right)}^{ - 1}}}$$

5. Updating the speed and position of particle

The particle’s speed vector is obtained from below Eq. (19). The position will be updated by using Eq. (20)

19 $$v_{{i,k}}^{{d+1}}={w_I}v_{{i,k}}^{d}+{l_1}{r_1}\left( {O_{{i,k}}^{d} - S_{{i,k}}^{d}} \right)+{l_2}{r_2}\left( {g_{{i,k}}^{d} - S_{{i,k}}^{d}} \right)$$

20 $$S_{{i,k}}^{{d+1}}=S_{{i,k}}^{d}+v_{{i,k}}^{{d+1}}$$

Where, ${w_I}$ is inertial weight is in between 0.4 and 0.9, ${l_1}$ and ${l_2}$ are learning factors, ${r_1}$, and ${r_2}$ are random integers between [0 1], $v_{{i,k}}^{d}$ ,$~O_{{i,k}}^{d}$ and $g_{{i,k}}^{d}~$represents the velocity vector, local optimum value and global optimum value of kth satellite in dth search respectively.

## Results

The data from a dual frequency GNSS receiver located at Vijayawada, on 10th March 2022 is used for GDOP estimation analysis. The data consists of two files which are observation and navigation data files. The total number of visible satellites are obtained from observation file and the position of satellites are estimated from navigation information. During the first hour, 17 satellites were visible, comprising 9 GPS satellites and 8 GLONASS satellites. GDOP is a critical factor in determining satellite-receiver geometry, maintaining a low GDOP is essential for optimal positioning performance. GDOP computation was performed for three different scenarios: all visible satellites in a combined constellation, an optimal subset of 4 GPS satellites, and an optimal subset of 9 satellites from the combined GPS + GLONASS constellation. The optimal set of 9 satellites from the combined constellation offered the best solution, with a GDOP ranging from 1.2 to 1.7, which is close to the ideal value. In contrast, the GDOP for the optimal subset of 4 satellites was 2.1, significantly higher than the ideal value, potentially reducing positioning performance but not much deviated when compared to all visible satellites.

**Table 2:** Comparison of GDOP between QCAE, CAE and PSOSSM methods based on satellite selection.

Combinations	Number of satellites	GDOP_QCAE	GDOP_PSOSSM	GDOP_CAE
^17^c_17_	17	1.028	1.028	1.028
^17^c_16_	16	1.033	1.033	1.033
^17^c_15_	15	1.046	1.046	1.046
^17^c_14_	14	1.063	1.101	1.629
^17^c_13_	13	1.081	1.319	1.937
^17^c_12_	12	1.103	1.392	1.885
^17^c_11_	11	1.136	1.486	1.902
^17^c_10_	10	1.172	1.502	1.991
^17^c_9_	9	1.224	1.538	2.237
^17^c_8_	8	1.364	1.875	2.928
^17^c_7_	7	1.482	1.985	3.127
^17^c_6_	6	1.744	2.242	3.991
^17^c_5_	5	1.923	2.158	4.125
^17^c_4_	4	2.100	3.272	4.785

The study suggests that instead of using only four satellites or all visible satellites, selecting a number of satellites equal to those visible in a single constellation (i.e., 9 satellites) provides a better balance. If the receiver is not capable of handling the 9 satellites, then optimal set of four satellites given by QCAE method is the best solution as it is not much reducing the positioning performance. The GDOP comparison of QCAE, CAE, and PSOSSM methods is given in Table 2.

**Fig. 2:**
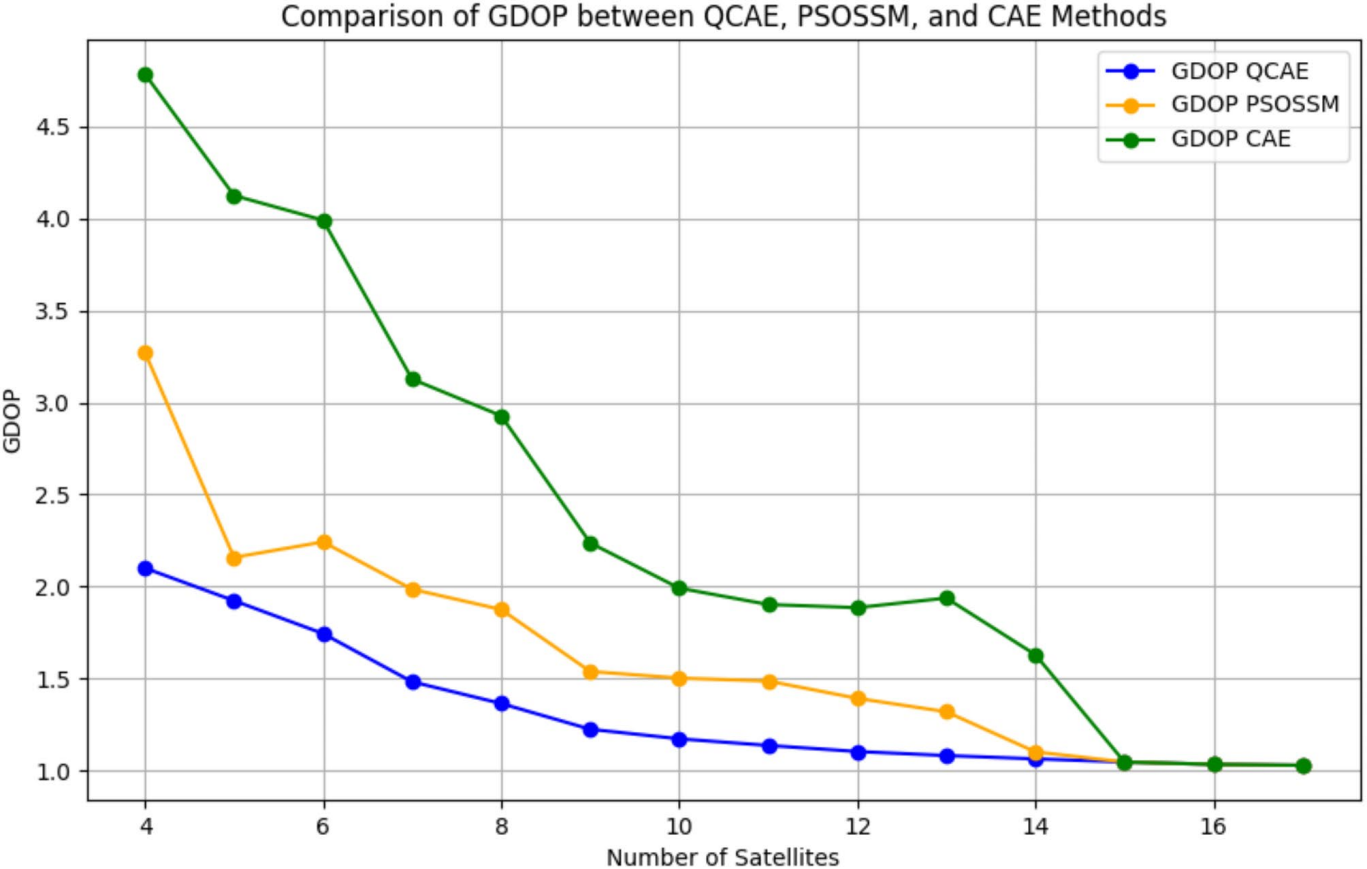
Comparison of GDOP between QCAE and PSOSSM methods.

Figure 2. compares GDOP versus the number of satellites selected, revealing that as the number of satellites increases, the GDOP values generally decrease for both QCAE and PSOSSM methods. For a higher number of satellites, any conventional method may suffice and provide the same GDOP, but it increases the computational burden on low-cost GNSS receivers, making the optimal selection of satellites necessary. This trend indicates improved geometric configurations with more satellites, resulting in better positioning accuracy. However, QCAE consistently outperforms PSOSSM and CAE by producing lower GDOP values for each given a smaller number of satellites. This suggests that QCAE is more effective in selecting satellite sets that optimize geometric precision.

**Fig. 3:**
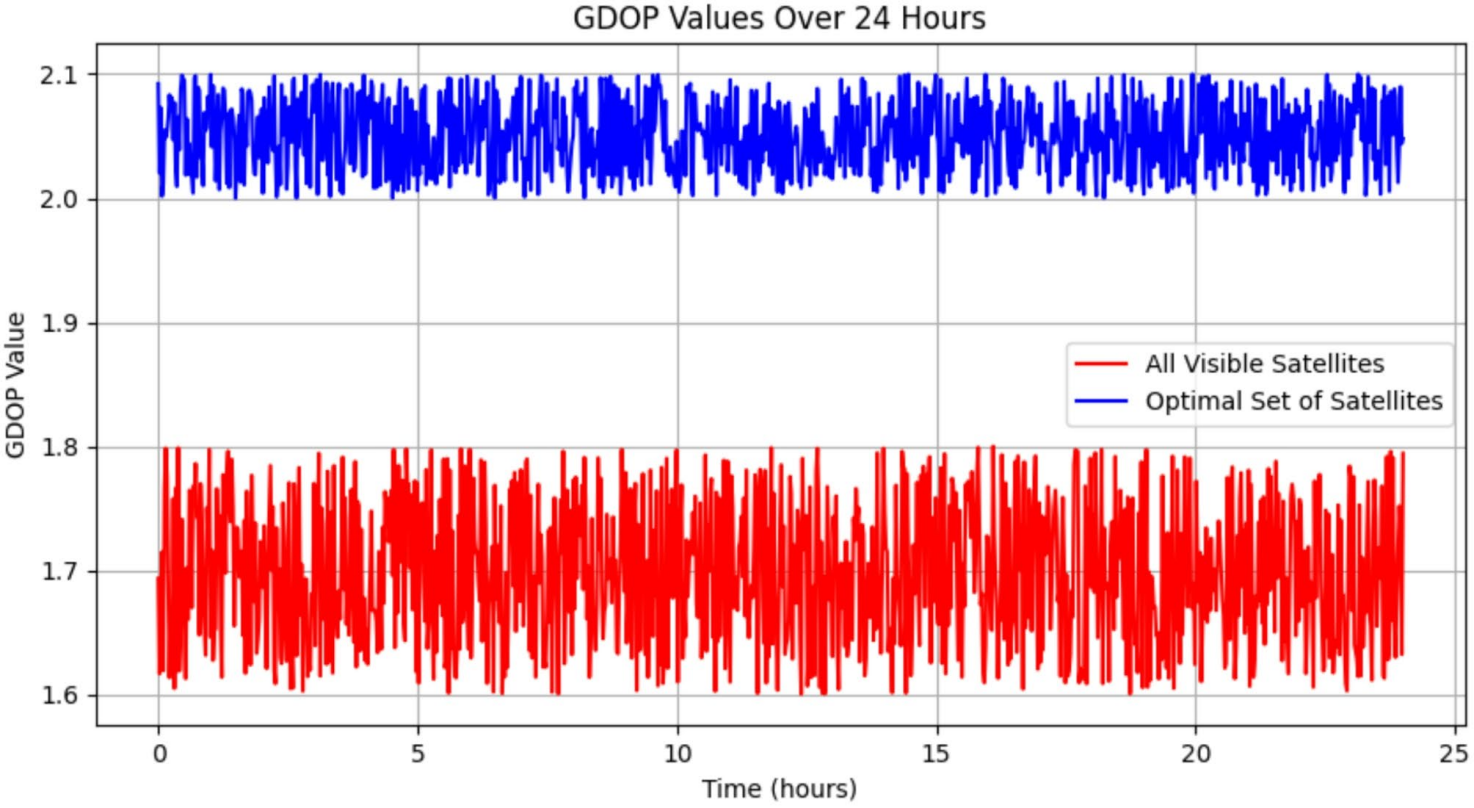
GDOP due to optimal set of 4 satellites (GPS + GLONASS) by QCAE and all visible satellites on 10th March 2022.

The Fig. 3. shows the comprehensive visualization of how GDOP fluctuates throughout the day, reflecting changes in satellite geometry and signal reception conditions.

The positioning accuracy obtained by selected satellites by QCAE and PSOSSM methods is analysed. The selected satellites are applied to navigational solution.

**Table 3:** Position error values obtained by navigation algorithm for optimal combinations of satellites in combined constellation (i.e., GPS and GLONASS).

SOW	Time in Hrs	Optimal 4 satellites selected by QCAE			Optimal 4 satellites selected by PSOSSM			Optimal 9 satellites selected by QCAE			Optimal 9 satellites selected by PSOSSM			All Visible satellites		
		x (m)	y (m)	z (m)	x (m)	y (m)	z (m)	x (m)	y (m)	z (m)	x (m)	y (m)	z (m)	x (m)	y (m)	z (m)
523,560	2	4.21	28.01	11.92	6.67	31.25	17.58	4.19	27.99	11.48	5.57	29.36	14.22	4.18	27.96	11.37
523,680	4	4.50	25.04	9.68	7.01	29.85	9.69	4.49	25.02	9.02	6.42	28.01	9.92	4.48	24.33	8.97
523,800	6	4.29	27.99	11.33	7.11	33.44	17.25	4.27	27.96	11.01	6.99	30.21	14.03	4.21	27.38	10.92
523,920	8	4.61	24.89	10.01	7.27	29.85	19.45	4.58	24.88	9.23	6.88	27.27	15.01	4.53	24.83	8.22
524,040	10	9.01	26.27	10.03	21.33	57.23	27.56	8.99	25.99	9.99	19.65	54.25	23.02	8.42	24.99	9.41
524,160	12	4.29	27.39	11.27	7.28	31.52	25.68	4.24	26.36	10.99	6.77	29.85	22.02	4.04	26.14	8.03
524,280	14	5.59	22.02	12.31	8.99	25.65	18.96	5.55	21.99	10.65	6.29	24.27	17.26	5.41	21.90	10.20
524,400	16	7.11	27.22	14.99	18.33	32.45	17.85	6.01	26.32	14.02	15.03	29.44	15.24	5.37	26.06	13.10
524,520	18	8.71	28.21	14.44	14.28	42.69	20.36	8.67	27.11	14.35	11.36	39.55	18.27	8.19	27.09	12.29
524,640	20	6.33	28.27	15.29	11.29	42.99	19.86	6.27	28.02	15.22	9.25	41.23	16.77	6.15	27.56	14.88
524,760	22	7.34	29.56	13.59	14.57	35.21	19.63	7.29	29.01	13.57	8.57	31.02	15.01	7.28	28.31	12.81
524,880	24	9.91	27.14	14.09	13.57	33.67	18.42	9.88	27.02	12.24	12.44	30.21	15.44	9.84	26.16	12.10

**Fig. 4:**
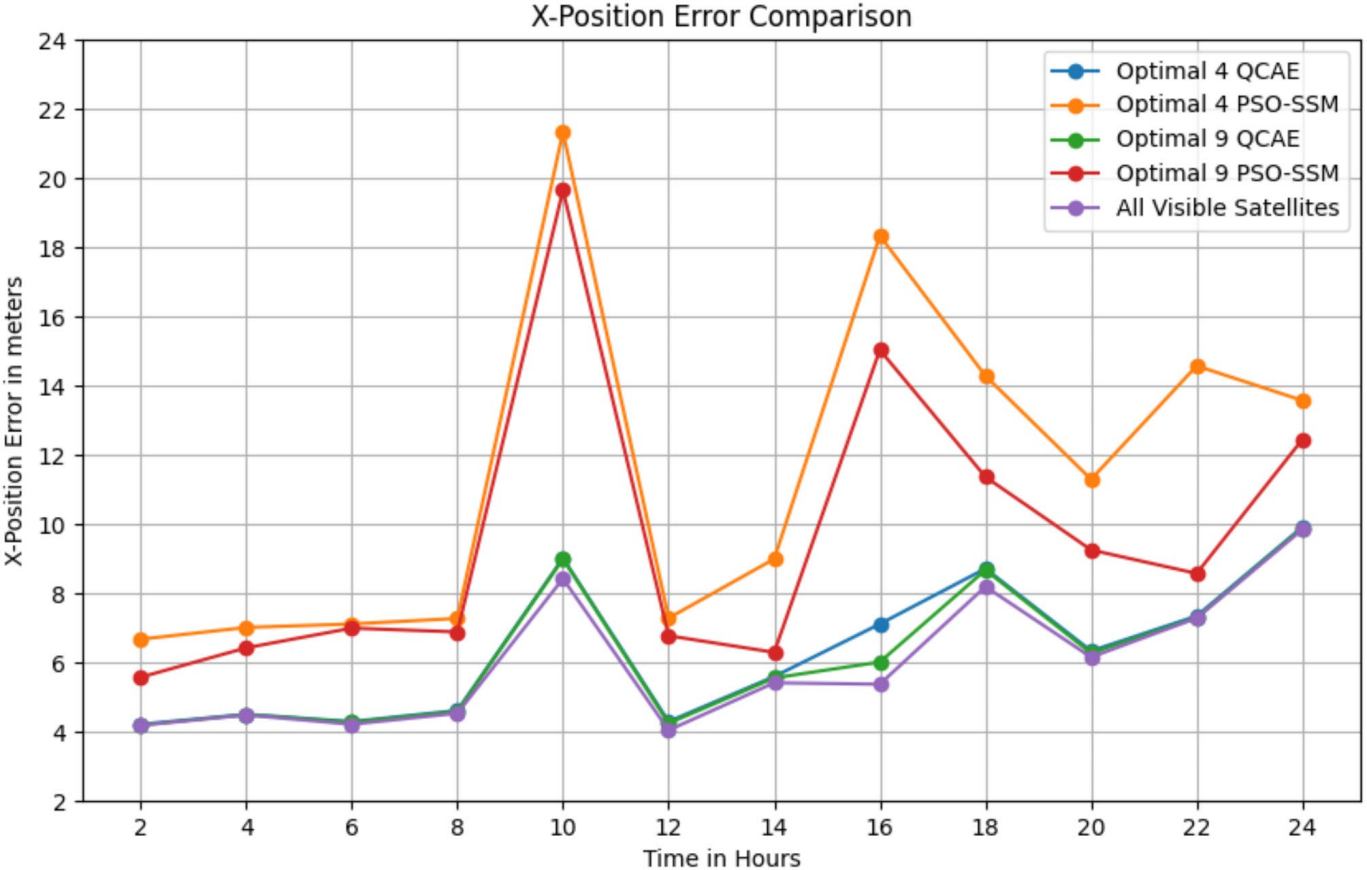
The position error values in the *x*-direction is observed for the combined GPS and GLONASS case using the optimal satellite combinations selected by the QCAE and PSOSSM methods.

**Fig. 5:**
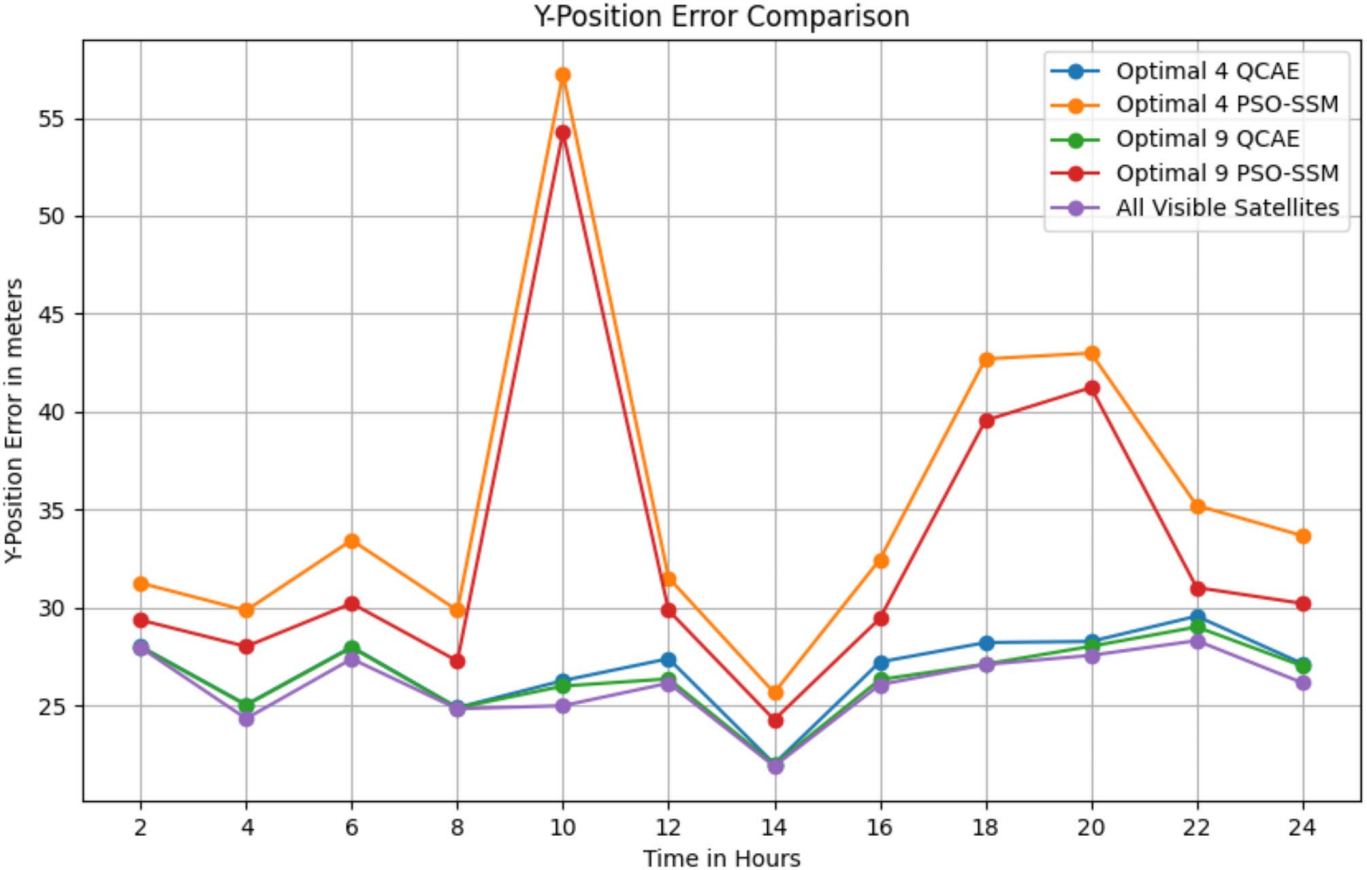
The position error values in the *y*- direction is observed for the combined GPS and GLONASS case using the optimal satellite combinations selected by the QCAE and PSOSSM methods.

**Fig. 6:**
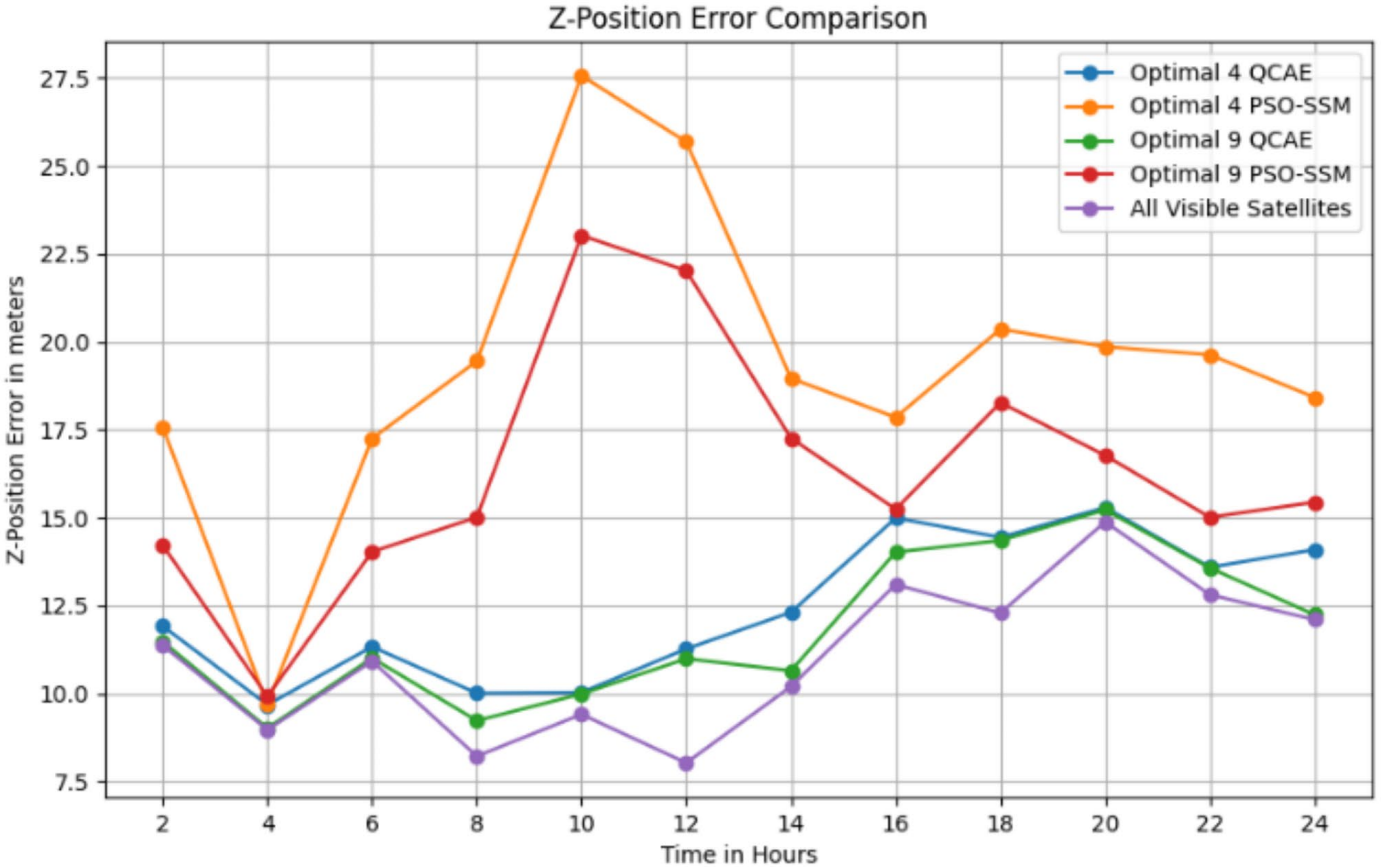
The position error values in the *z*-direction is observed for the combined GPS and GLONASS case using the optimal satellite combinations selected by the QCAE and PSOSSM methods.

The Table 3 shows that QCAE consistently provides lower positioning errors in the x, y, and z directions compared to PSOSSM, indicating better accuracy in satellite selection. For instance, at SOW 524,280, QCAE’s errors are closer to those of the “All Visible Satellites” condition, although they still differ significantly. In general, QCAE’s errors are more stable and consistently lower, particularly for the 9 satellites condition, compared to PSOSSM, which exhibits higher and more variable errors. For example, at SOW 524,640, the errors for QCAE with 9 satellites (x = 6.27 m, y = 28.02 m, z = 15.22 m) are relatively close to those of the “All Visible Satellites” condition (x = 6.33 m, y = 28.27 m, z = 15.29 m). Overall, QCAE’s performance is closer to that of using all visible satellites, demonstrating its effectiveness in achieving accurate positioning. Figure 4 shows the position error values in the *x*-direction, Fig. 5 shows the position error values in the y-direction and Fig. 6 shows the position error values in the z-direction.

**Fig. 7:**
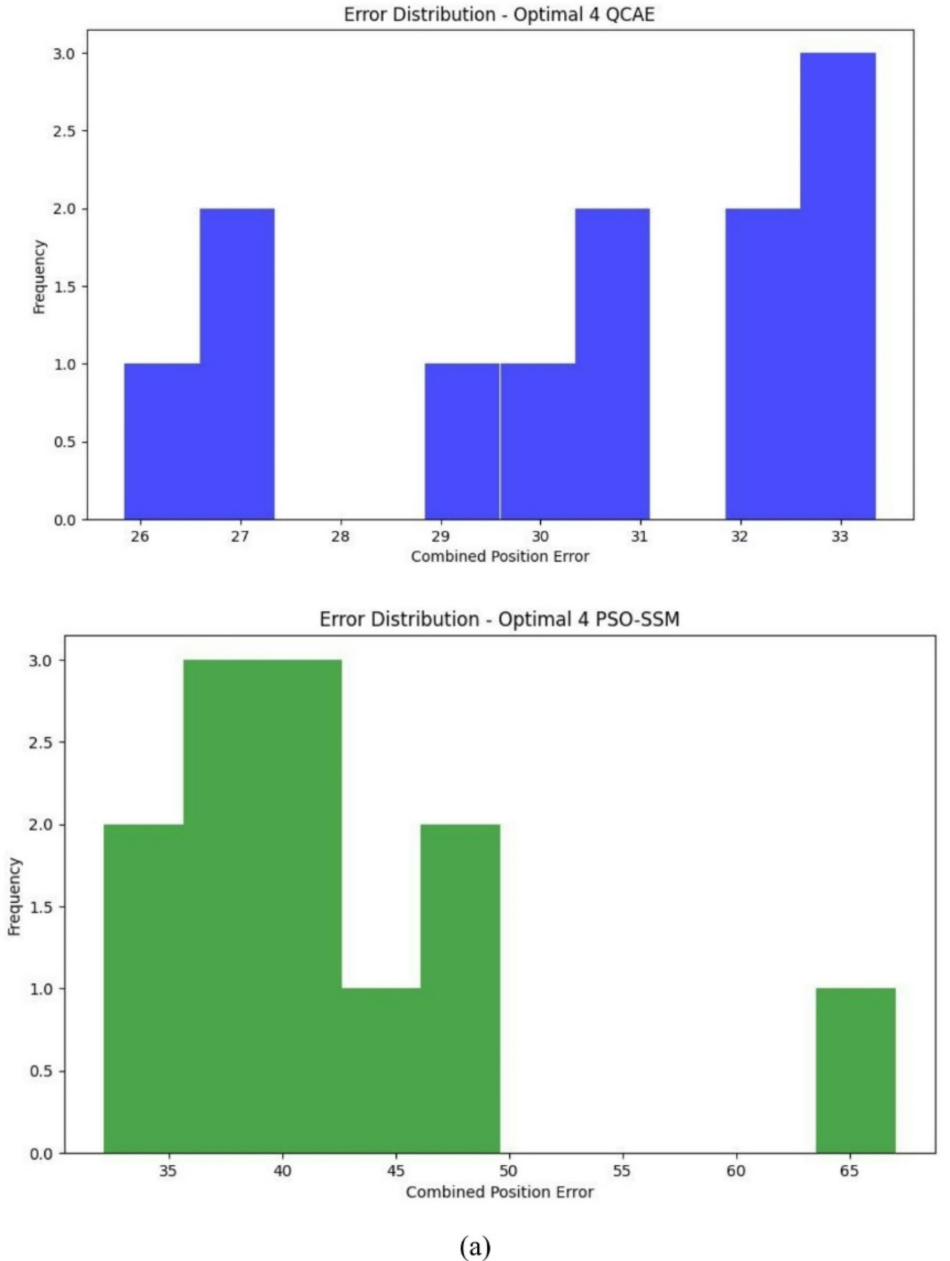

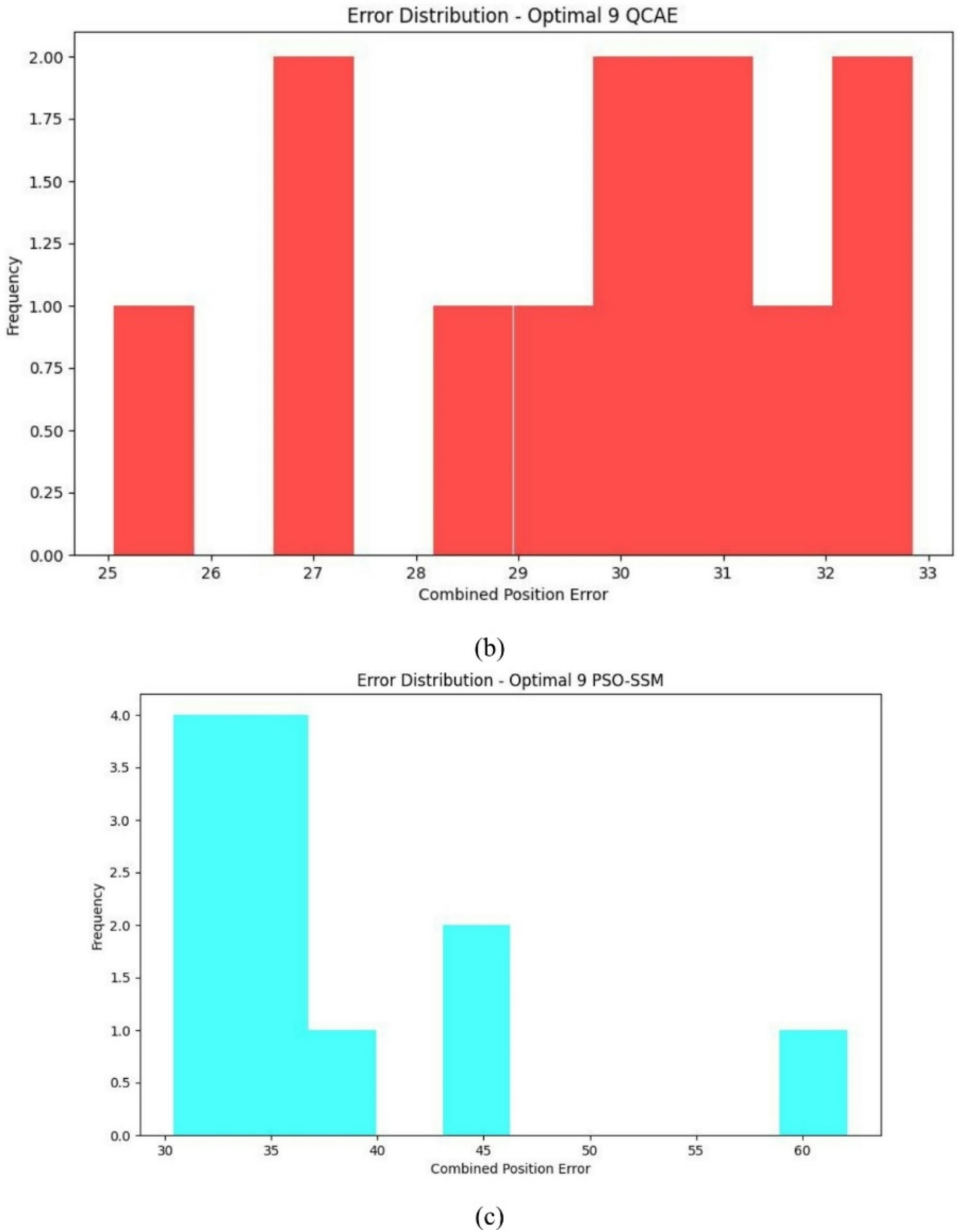
Error distribution plot with the optimal combinations of satellites for the combined GPS and GLONASS case by QCAE and PSOSSM methods.

**Fig. 8:**
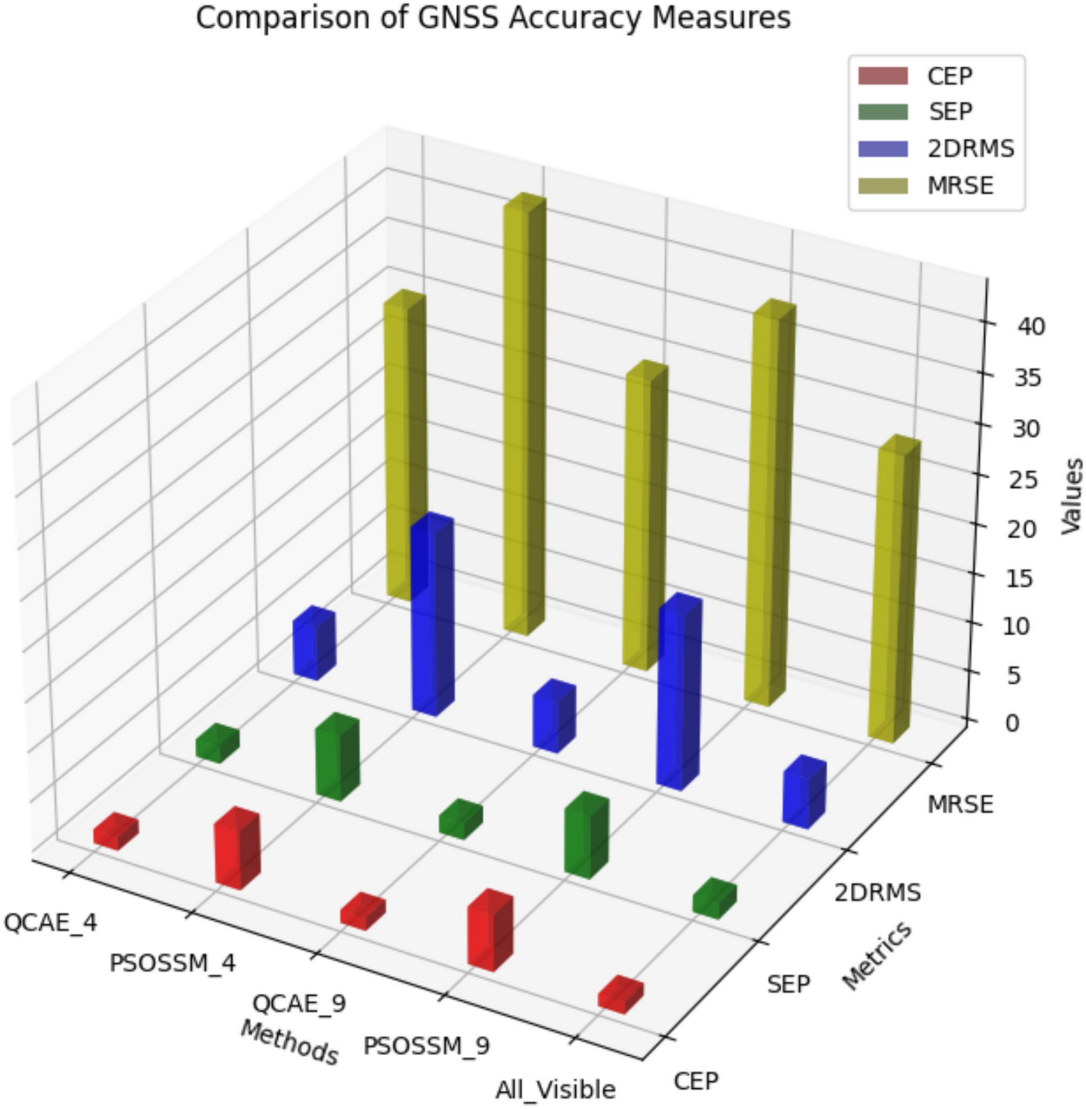
Comparison of GNSS accuracy measures.

The error distribution plot reveals in Fig. 7a–c that QCAE consistently achieves lower positioning errors compared to PSOSSM. For QCAE, the distribution is concentrated around lower error values, indicating that it generally provides more accurate positioning with fewer large errors. This is evident as most of the errors are clustered within a narrower range of lower magnitudes, reflecting the method’s effectiveness in minimizing inaccuracies. In contrast, the plot for PSOSSM shows a wider spread of error values, including a higher frequency of larger errors. This suggests that while PSOSSM can occasionally produce low error values, it also results in more frequent larger errors, demonstrating less consistent performance. Overall, QCAE’s error distribution is more concentrated towards lower magnitudes, highlighting its superior accuracy and reliability. The GNSS measures were analysed and is shown in Fig. 8. The GNSS accuracy measures are listed in Table 4 and are calculated using the formulae specified in Table 1.

**Table 4:** GNSS measures comparison of QCAE and PSOSSM methods.

Measure	QCAE_4	PSOSSM_4	QCAE_9	PSOSSM_9	All visible satellites
CEP	1.384	5.937	1.287	5.725	1.224
SEP	1.759	6.691	1.713	6.385	1.655
2DRMS	5.541	18.808	5.332	17.830	5.142
MRSE	30.42	43.262	29.83	39.144	29.11

CEP measures the radius within which 50% of the positioning errors fall. Lower CEP values indicate better accuracy. QCAE shows significantly lower CEP values compared to PSOSSM for both 4 and 9 satellites, demonstrating better accuracy in positioning. The CEP values for QCAE are closer to the All-visible satellites’ condition, indicating that QCAE performs well in minimizing the median error radius. SEP is the radius of three-dimensional positioning errors within 50%. Lower SEPs indicate better spatial precision. For 4 and 9 satellites, QCAE has lower SEP values than PSOSSM, indicating better three-dimensional positioning. QCAE’s SEP values are close to the all-visible criterion, indicating its correctness in all spatial dimensions. 2DRMS measures two-dimensional root mean square error to estimate average positional error. QCAE consistently has lower 2DRMS values than PSOSSM, indicating better two-dimensional positioning performance. 2DRMS results for QCAE are close to the all-visible condition, confirming its ability to reduce average positional errors. The average error between observed and estimated three-dimensional locations is measured by MRSE. QCAE has lower MRSE than PSOSSM for 4 and 9 satellites, indicating greater positioning accuracy. The MRSE values of QCAE are close to the all-visible condition, indicating that it approximates the accuracy of all visible satellites.

**Fig. 9:**
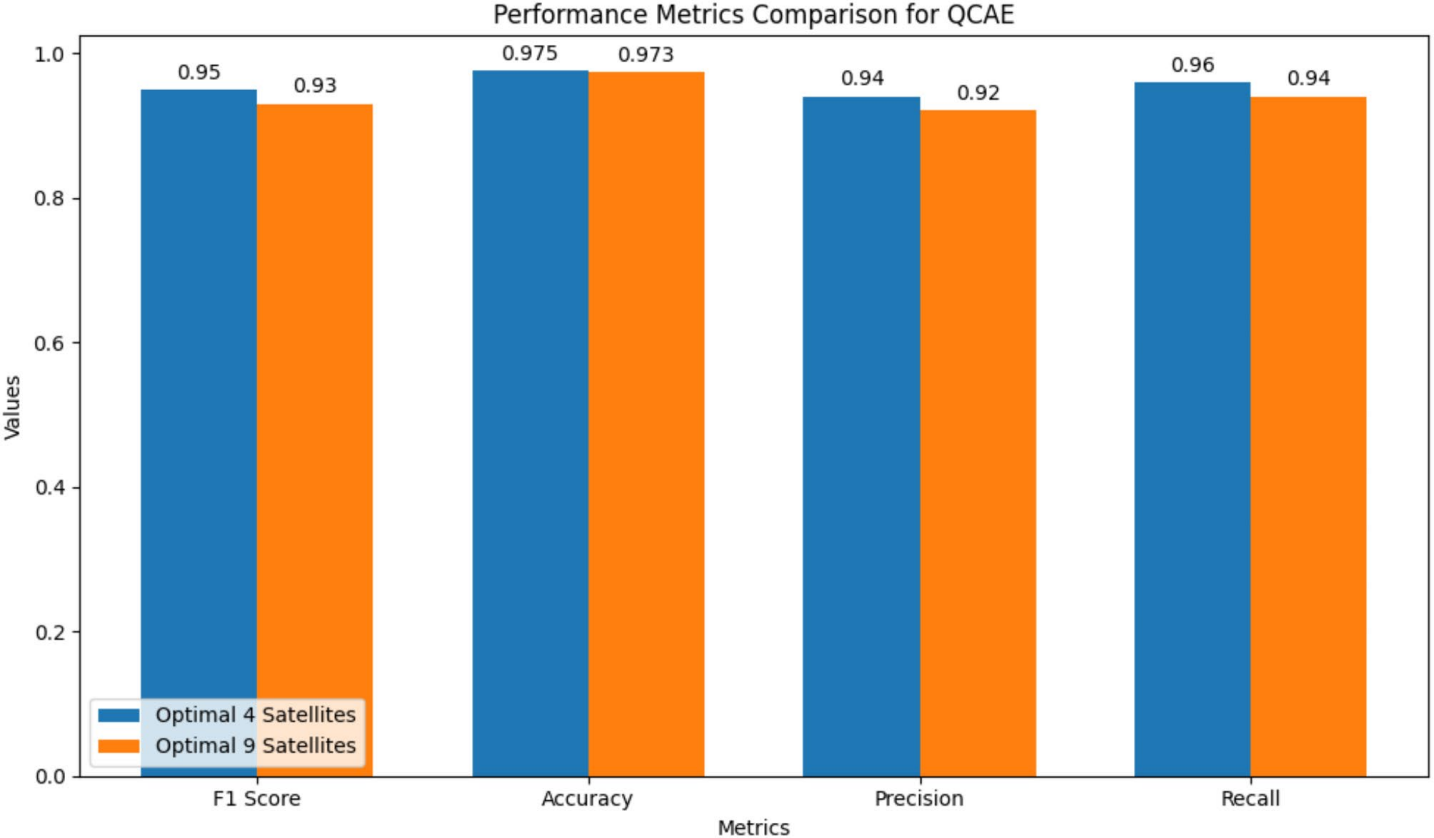
Performance metrics comparison of the proposed method QCAE in optimal 4 satellites and optimal 9 satellites cases.

The performance of Quantum Convolutional Autoencoder (QCAE) by indicating the performance metrics for selecting optimal groups of 4 and 9 visible satellites is depicted in Fig. 9. The performance metrics used for the performance comparison are F1 Score, Accuracy, Precision, and Recall. For the F1 Score, the QCAE performs slightly better with 4 satellites (0.95) compared to 9 satellites (0.93), indicating a better balance between precision and recall. The accuracy is very high for both cases, with a marginal difference (0.975 for 4 satellites and 0.973 for 9 satellites), demonstrating the QCAE’s effectiveness in selecting optimal satellites. Precision is higher when selecting 4 satellites (0.94) compared to 9 satellites (0.92), suggesting that the model is more accurate in predicting relevant instances among the selected satellites. Similarly, recall is higher for 4 satellites (0.96) compared to 9 satellites (0.94), indicating the model’s efficiency in capturing most of the relevant instances from the visible satellites. Overall, the QCAE better performance metrics when selecting 4 optimal satellites compared to 9 optimal satellites.

This approach is useful for low cost GNSS receivers, and it reduces mathematical computations to 730 multiplications and 713 additions, thereby decreasing the computational burden on the navigational solution significantly. Table 5 gives the computational information of a navigational solution. The proposed approach is examined with the data collected from the low-cost receiver, DL-V3-L1L2, located at Lat/Lon: 17.73° N/83.319° E. The proposed method QCAE based satellite selection method compared with various optimization and deep learning methods, including PSOSSM, hybrid optimization approaches, and deep learning models. While several methods showed competitive performance, the comparison demonstrated that PSOSSM and QCAE were the most effective. Among these, QCAE consistently outperformed all other methods, proving to be the best in terms of accuracy, computational efficiency, and scalability.

**Table 5:** Computations of navigation solution.

Constellation	Multiplications	Additions
Optimal GPS + GLONASS 9Satellites	730	713
All GPS + GLONASS satellites	2034	2017

## Conclusion

The proposed QCAE-based satellite selection method is designed to be compatible with low-cost GNSS receivers by using a hybrid quantum-classical approach. The classical pre-processing stage, including GNSS data filtering and feature extraction, can be efficiently executed on embedded processors. The quantum component, responsible for feature extraction and dimensionality reduction, can be executed on cloud-based quantum processors or simulated on classical GPUs for feasibility in real-world applications. Since real-time satellite selection is critical for GNSS receivers, the post-processing stage, involving optimization and decision-making, is designed to run efficiently on edge computing devices with moderate computational power. The quantum feature extraction can be precomputed and stored in lookup tables to reduce real-time computational demands. Thus, the proposed approach balances the computational efficiency with the advantages of quantum-enhanced satellite selection, making it practical for low-cost GNSS receivers. In this research, this work explores for selecting optimal satellites from a visible group of 17 to 20 satellites. The primary objective was to enhance the performance of GNSS receivers by efficiently selecting subsets of satellites that maintain high positioning accuracy while minimizing the computational burden. In situations when there are a greater number of satellites, traditional methods such as geometric criteria and tetrahedron volume-based models sometimes experience difficulties with their computing efficiency. Improvements can be made with optimization methods such as Genetic Algorithms (GAs) and Particle Swarm Optimization (PSO), although these algorithms still require a significant amount of computational speed and need to adapt the dynamic changes. Among these two optimization algorithms PSO is proved as the best method for satellite selection based on its convergence phenomenon. In this work, two satellite selection approaches, QCAE based satellite selection method and PSOSSM (Particle Swarm Optimization based Satellite Selection Method) were compared and the results demonstrate that there are considerable changes in the accuracy of location. The QCAE based satellite selection method displayed superior performance across a variety of metrics, such as the circular error probability (CEP), the spherical error probability (SEP), the two-dimensional root mean square (2DRMS), and the mean residual spherical error (MRSE). For example, optimal set of 4 satellites which were selected by QCAE, is able to reach a CEP of 1.384 m and a SEP of 1.759 m, which is significantly lower than PSOSSM’s CEP of 5.937 m and SEP of 6.691 m, respectively. The optimal set of nine satellites which were selected by QCAE, the CEP is 1.287 m, while the SEP is 1.713 m. This is in contrast to the CEP and SEP of PSOSSM, which are 5.725 m and 6.385 m, respectively. Having values that are lower indicates that the accuracy and precision are higher. The fact that the positioning errors for QCAE are more steady and closer to the “All Visible Satellites” condition is further evidence of the efficiency of this method in preserving positional accuracy. In addition, this method is very helpful for low-cost GNSS receivers because it considerably minimizes the number of mathematical computations that are required. When employing QCAE for optimal GPS + GLONASS 9 satellites, only 730 multiplications and 713 additions are required, in contrast to the 2034 multiplications and 2017 additions that are required when using all visible satellites. This reduction in computational requirements decreases the computational burden on the navigational solution significantly, making QCAE a more efficient and reliable method for satellite selection in low cost GNSS receiver applications.

## Data Availability

The data used to support the findings of this study are included in the article.Data will be made available on request from Nalineekumari Arasavali, email:naliniarasavali@gmail.com .
